# Non-neoplastic and neoplastic scrotal pathologies on magnetic resonance imaging: a pictorial essay

**DOI:** 10.1590/0100-3984.2024.0073-en

**Published:** 2025-01-06

**Authors:** Thiago de Oliveira Caetano, Alice Schuch, Ivan Morzoletto Pedrollo

**Affiliations:** 1 Hospital Moinhos de Vento, Porto Alegre, RS, Brazil

**Keywords:** Magnetic resonance imaging, Scrotum, Seminoma, Ressonância magnética, Escroto, Seminoma

## Abstract

Magnetic resonance imaging is an essential tool for the assessment of the scrotum, particularly in cases with inconclusive ultrasound findings. It has a great capacity to differentiate between intratesticular and extratesticular lesions, as well as between neoplastic and non-neoplastic lesions. By providing an accurate characterization of lesions, magnetic resonance imaging plays a crucial role in preoperative tumor staging and decision-making. This pictorial essay highlights the key non-neoplastic and neoplastic testicular pathologies, as evaluated by magnetic resonance imaging. The recognition of these pathologies underscores the role the radiologists play in the care of patients with scrotal lesions, by providing an appropriate evaluation of the relevant imaging characteristics.

## Abstract (pt)

A ressonância magnética é uma ferramenta importante na avaliação da bolsa testicular em casos com achados inconclusivos pela ultrassonografia. A capacidade da ressonância magnética de diferenciar entre lesões intratesticulares e extratesticulares, bem como entre condições neoplásicas e não neoplásicas, tem papel crucial na caracterização de lesões, no estadiamento de tumores e auxilia na decisão pré-operatória. Este ensaio apresenta imagens de ressonância magnética que destacam as principais patologias não neoplásicas e neoplásicas. O reconhecimento dessas patologias reforça o papel do médico radiologista na linha de cuidado de pacientes com lesões da bolsa testicular mediante uma avaliação adequada das suas características de imagem.

## INTRODUCTION

Because it is a low-cost technique that is widely available, does not use ionizing radiation, and can generate images in real time, ultrasound is the first-line imaging modality for evaluating the scrotum. However, it has limitations, mainly due to its relatively small field of view and the fact that it is operator dependent. Changes in tissue echogenicity can be nonspecific, which limits the characterization of possible lesions^(1-3)^.

**Figure 1:**
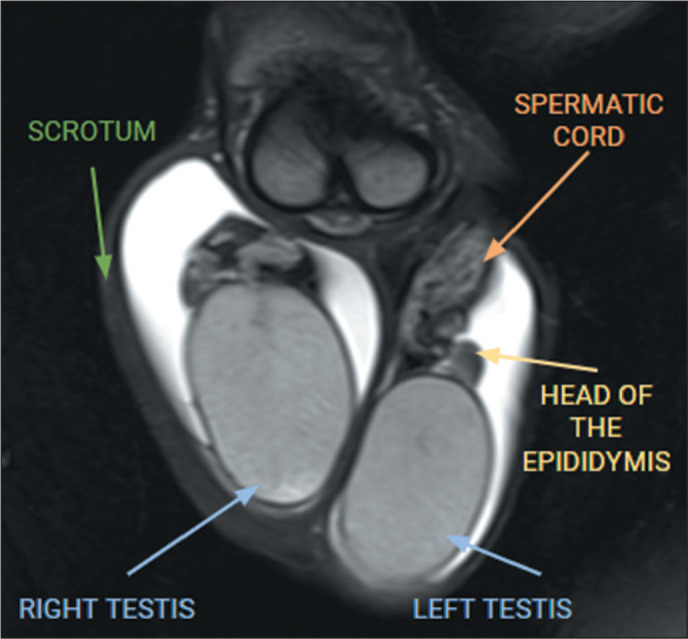
MRI showing the normal anatomy of the scrotum on coronal T2- weighted images with fat saturation. The normal adult testis is a homogeneous oval structure that is hyperintense on T2-weighted images, hypointense to isointense on T1-weighted images, and surrounded by the tunica albuginea, which is hypointense on T1- and T2-weighted images^(2,3,5,7)^. The epididymis is isointense relative to the testis on T1-weighted images, hypointense on T2- weighted images, and best seen on sagittal T2-weighted images^(1-3,7)^. The testis and epididymis gradually enhance after intravenous administration of contrast medium, probably because of the integrity of the blood-testicular barrier^(2,5,7)^. On the highest b value of diffusion-weighted imaging and on the apparent diffusion coefficient map, the testicular parenchyma is usually visualized as hyperintense and slightly hypointense, respectively, because of the complex histology of its parenchyma^(2,3)^.

**Figure 2:**
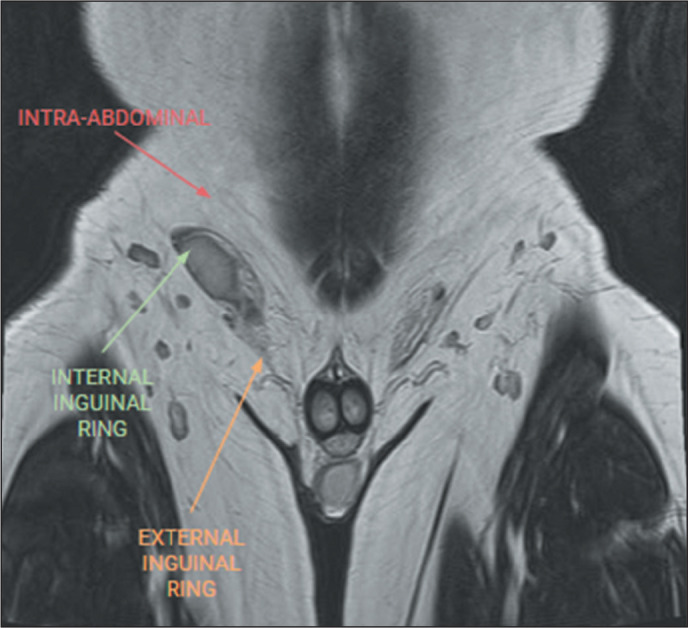
Undefined right testicle in the scrotum, visualized in the right inguinal canal. The arrows in the right inguinal region demonstrate the potential locations of an ectopic testicle. The most common location is in the internal inguinal ring, followed by the external inguinal ring and the intra-abdominal region^(8)^.

Magnetic resonance imaging (MRI) of the scrotum, as shown in Figure 1, is indicated for cases in which ultrasound is inconclusive, in the following scenarios^(1,3,4)^: characterization of and differentiation between intratesticular and paratesticular lesions; differentiation between germ cell and sex cord tumors; staging of the site of testicular malignancies; differentiation between seminomatous and nonseminomatous tumors; evaluation of testicular trauma; and evaluation of cryptorchidism (Figure 2).

This pictorial essay aims to present the aspects of the main non-neoplastic and neoplastic pathologies of the scrotum seen on MRI.

## NORMAL ANATOMY OF THE SCROTUM ON MRI

The image characteristics of the scrotum, with the description of the main anatomical structures (testes and epididymis), are demonstrated in Figure 1^(2,3,5,6)^.

## NON-NEOPLASTIC PATHOLOGIES

### Infection (epididymitis and epididymo-orchitis)

Epididymitis and epididymo-orchitis are common causes of acute testicular pain, usually due to retrograde infection of the lower urinary tract^(2,5,7)^. In cases of infection with suspected complications, such as abscess formation, venous infarction, and pyocele (due to rupture of the tunica vaginalis), MRI plays a complementary role^(2,5)^.

**Figure 3:**
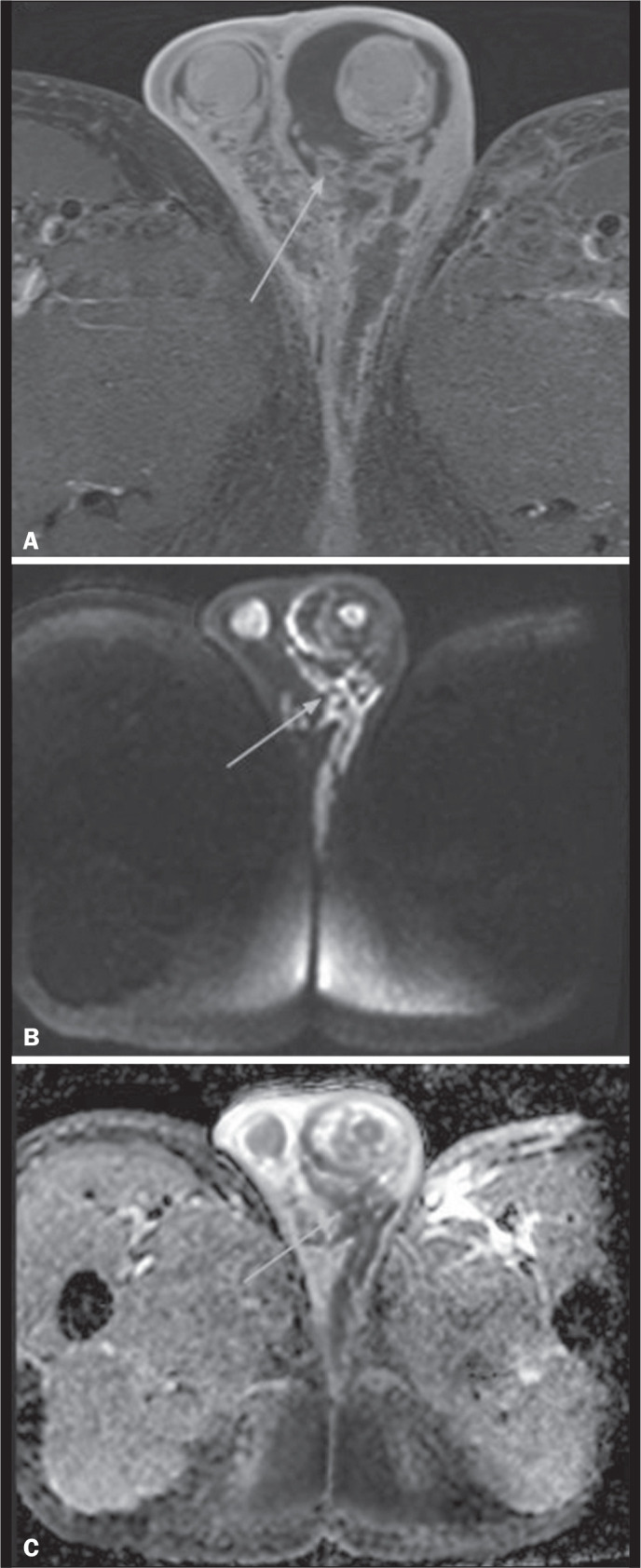
Abscesses and pyoceles. Contrast-enhanced axial T1-weighted sequence with fat suppression showing a pyocele (A) with restricted diffusion of the content (B,C), and with a multiloculated, septated appearance, extending to the right hemi-scrotum and to the subcutaneous tissue. Abscesses present with hyperintense central content on T2-weighted images, restricted diffusion, and peripheral contrast enhancement. Edematous infiltration of adjacent soft tissues is typical, with hyperintense areas on T2-weighted images with fat suppression(2,5).

On MRI, the epididymis appears enlarged, edematous and with early enhancement, as demonstrated in Figure 3. This method can also aid in the detection of perianal fistulas associated with testicular abscesses, which manifest as linear hypointense structures on T1-weighted imaging and hyperintense on T2-weighted images with fat suppression^(5)^.

### Testicular infarction

Testicular infarction is rare and can be complete or segmental. Patients with testicular infarction present with severe testicular pain^(2,3)^. Complete testicular infarction is typically associated with testicular torsion. In contrast, segmental testicular infarction has various established causes, including trauma, acute epididymo-orchitis, and hematologic disorders (such as sickle cell disease and vasculitis). Segmental infarctions can be confused with expansile lesions and are a major cause of a finding of testicular pseudotumor^(2)^.

**Figure 4:**
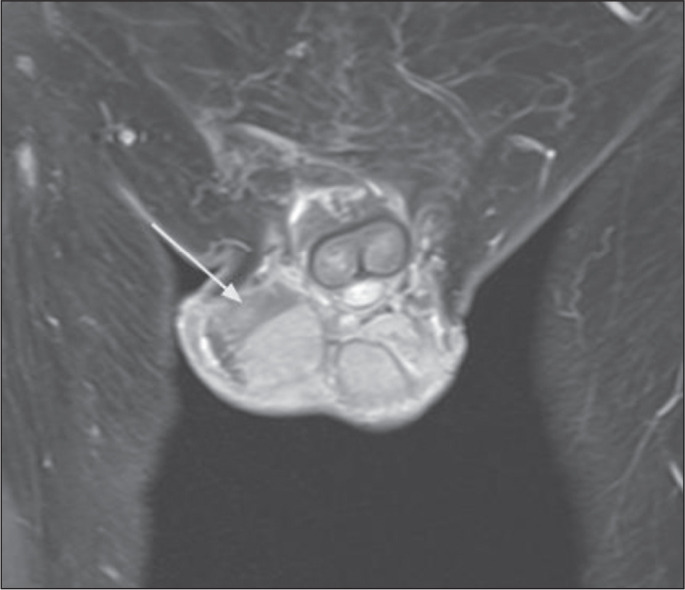
Small segmental testicular infarction. Contrast-enhanced coronal T1weighted image with fat suppression, showing a band-like unenhanced area in the upper pole of the right testicle (arrow).

On MRI, testicular infarction is confirmed by the absence of contrast enhancement of ischemic tissue. The presence of a triangular-shaped intratesticular area without contrast enhancement, pointing toward the rete testis, with a hypointense signal on T2-weighted images and a contrast-enhanced rim (Figure 4), is strongly suggestive of segmental testicular infarction^(3)^. Sequences employing the post-contrast subtraction technique can be useful in this evaluation.

## NEOPLASTIC PATHOLOGIES

### Extratesticular lesions

For extratesticular lesions, MRI allows their precise localization and defines their anatomical relationships with adjacent structures^(1,2)^. Extratesticular solid neoplasms are rare. The most common extratesticular tumor is lipoma, followed by adenomatoid tumor^(1)^.

### Adenomatoid tumor

The most common benign tumor of the epididymis, accounting for 30% of all extratesticular tumors, is adenomatoid tumor. They are of mesodermal origin and can occur in the spermatic cord or tunica albuginea, where they can grow toward the testicular parenchyma, mimicking germ cell tumors^(2,3,5)^.

Adenomatoid tumors occur at different ages, most arising in individuals between 20 and 25 years of age, and are smooth, round, well-circumscribed lesions, varying in size from a few millimeters to 5 cm^(2)^.

**Figure 5:**
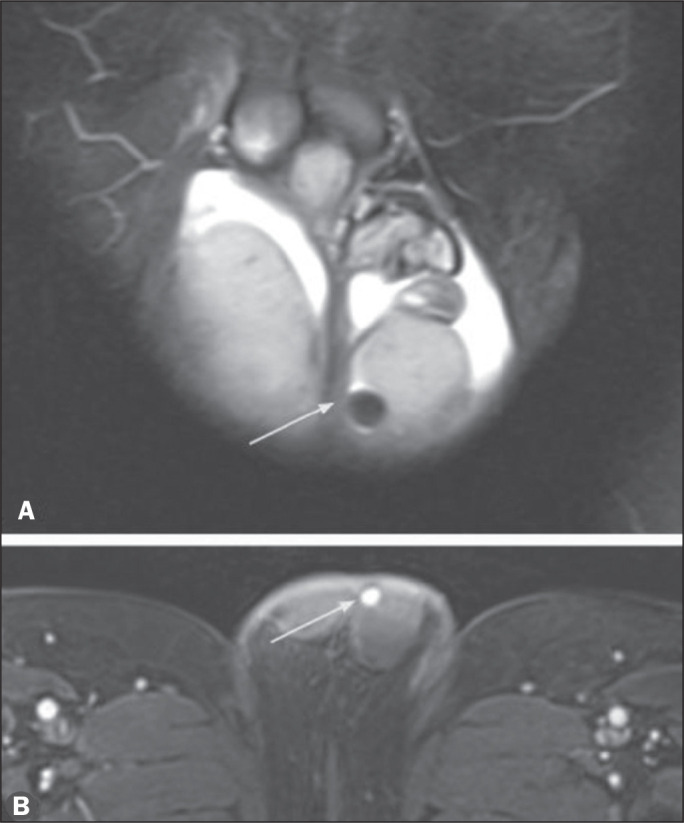
Left-sided adenomatoid tumor. Coronal T2-weighted image with fat suppression (A) and contrast enhanced T1-weighted image with fat suppression (B), showing a homogeneous, hypointense nodular extratesticular lesion (in A) with marked hypervascular enhancement (in B), findings characteristic of an adenomatoid tumor, which was confirmed after surgical resection.

It has been demonstrated that MRI is useful in distinguishing an extratesticular neoplasm from an intratesticular mass in the periphery of the testis^(2,4)^. The imaging findings are shown in Figure 5.

### Liposarcoma

**Figure 6:**
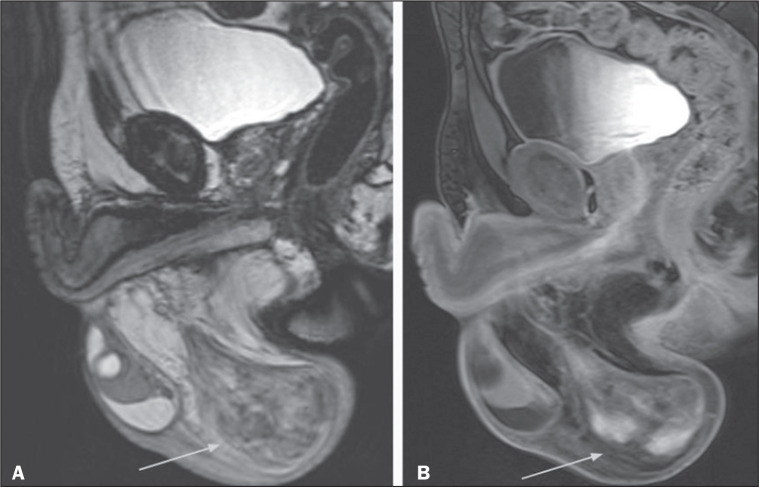
Liposarcoma of the scrotum. Sagittal T2-weighted image without fat suppression (A) and contrast-enhanced sagittal T1-weighted image with fat suppression (B), showing a large nodular lesion in the subcutaneous tissue of the right scrotum, with solid areas of high signal intensity on the T2-weighted image and early contrast enhancement (B), interspersed with areas of fat, related to liposarcoma of the scrotum, which was confirmed after surgery. Macroscopic fat can be identified as high signal intensity on T1- and T2-weighted images and low signal intensity on fatsuppressed sequences^(2,4,5)^. In addition, chemical shift artifacts can be observed at the interface between the soft tissues and the intratumoral fat components, as can heterogeneous contrast enhancement^(2,4)^.

Paratesticular liposarcomas are rare, accounting for only 7-10% of all intratesticular tumors. They arise from mesenchymal cells adjacent to the spermatic cord and are composed of adipose tissue and containing other types of tissue^(2)^. The average age at presentation of a paratesticular liposarcoma is 56 years (range, 50-70 years); they can be confused with inguinal hernias, hydroceles, or even benign or malignant tumors of the testicle or epididymis^(2)^. The imaging findings and characteristics are shown in Figure 6.

### Intratesticular lesions

Testicular cancer accounts for 1.0-1.5% of all malignant neoplasms in men, being most common in boys and young adult men (range, 15-34 years of age), and usually manifests as a painless testicular mass^(2,3)^.

Testicular tumors are classified as one of two types^(2-4,8)^: germ cell tumors (GCTs) and non-germ cell tumors (NGCTs). The majority (95%) of testicular neoplasms are GCTs, originating from the germinal epithelium and seminiferous tubules, and are divided fairly evenly between seminomatous and nonseminomatous tumors. Fewer than half of all GCTs are composed of a single cell type, and approximately 50% of those are seminomas, which are most commonly observed in individuals between 40 and 50 years of age^(3)^. Table 1 presents the main MRI findings of intratesticular tumors.

**Table 1:** Most common MRI findings in intratesticular tumors^(3,10)^.

Tumor type	Age (years)	T1WI	T2WI	Enhancement	Washout	Diffusion	Other features
GCTs Seminoma Nonseminomatous Epidermoid cyst NGCTs Leydig cell tumor Sertoli cell tumor Lymphoma	30-40 20-30 Prepubertal 5-10 20-30 5-10 20-30 > 60	Hypointensity Heterogeneous Hypointensity halo and central area of hyperintensity (“target”) Hypointensity Hypointensity Hypointensity	Hypointensity Heterogeneous Mixed signal intensity: hyperintensity and hypointensity (“onion skin” sign) Hypointensity Variable (hyperintensity or hypointensity) Hypointensity	Variable (mostly homogeneous) Heterogeneous None Hypervascular Hypervascular Markedly heterogeneous	No Variable - Yes Yes -	Restricted Restricted - Restricted Restricted Markedly restricted	Heterogeneous enhancement (fibrous septa); when larger, potentially overlapping with nonseminomatous features Necrosis, bleeding, cystic degeneration Teratoma without malignant potential; encapsulated oval lesion, lined by squamous epithelium containing keratin (typical appearance) Usually bilateral; can infiltrate the epididymis, spermatic cord, or skin

*Note:* Imaging findings can vary in testicular lesions and cannot be used in isolation to define histological subtypes. Mixed testicular lesions can exhibit characteristics of each subtype, making their differentiation even more difficult.

Among nonseminomatous GCTs, there are four basic, histologically diverse types^(2,3,8)^: embryonal carcinoma, teratoma, choriocarcinoma, and yolk sac tumor. Nonseminomatous GCTs typically occur earlier in life, between 30 and 40 years of age^(3)^.

**Figure 7:**
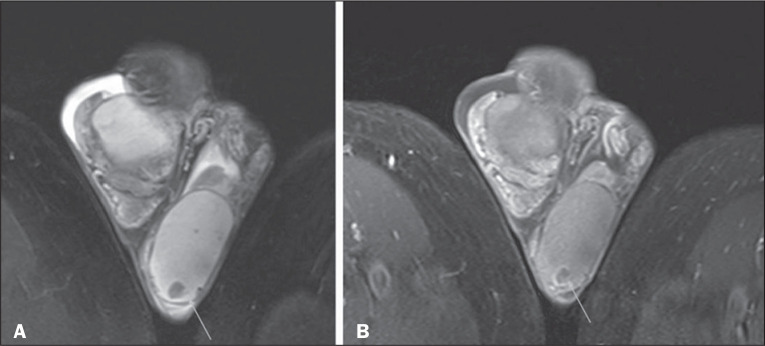
Leydig cell tumor. Coronal fatsaturated T2-weighted image (A) and dynamic contrast-enhanced sequence (B, delayed phase), showing a solid nodule in the lower pole of the left testis, with low signal intensity on the T2-weighted image (A), early enhancement on a dynamic contrast-enhanced sequence (image not available), and washout in the delayed phase (B).

All NGCTs are derived from the cells that form the sex cords (Sertoli cells) and the interstitial stroma (Leydig cells), with different incidences between age groups: 4% in adults and 10-30% in children^(3)^. Leydig cell tumors are the most common, and the typical presentation on MRI is that of a well-defined nodular lesion that is markedly hypervascular with low homogeneous signal intensity on T2-weighted images (Figure 7).

**Figure 8:**
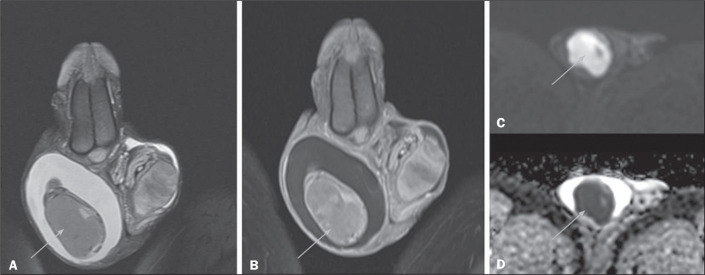
Testicular lymphoma. Coronal T2-weighted image with fat suppression (A), contrast-enhanced coronal T1-weighted image (B), and diffusion-weighted images (C,D), showing an expansile lesion occupying practically the entire right testicular parenchyma, with intermediate signal intensity on the T2-weighted image (A), heterogeneous contrast enhancement, and pronounced restricted diffusion (C,D).

Testicular lymphoma accounts for 1-9% of all testicular neoplasms and is the most common testicular neoplasia in patients over 60 years of age, most commonly having a bilateral presentation^(3)^. It can present as infiltrative, with invasion of adjacent structures (the most common form), together with lymph node enlargement, or even as a focal nodular lesion. It usually presents with intermediate or low signal intensity on T2-weighted images, homogeneous, pronounced restricted diffusion on diffusionweighted imaging, and a hypovascular enhancement pattern^(3)^, as depicted in Figure 8.

### Seminomatous and nonseminomatous GCTs

Conventional imaging criteria for identifying malignant lesions in the testis include a lesion that is predominantly hypointense relative to normal tissue on T2weighted images or that is heterogeneous on T2-weighted images with heterogeneous enhancement after intravenous contrast administration^(3)^. The presence of hemorrhage or necrosis within the tumor is considered a secondary sign of malignancy, as is extension of the tumor into the testicular tunics, paratesticular space, or spermatic cord^(3)^.

**Figure 9:**
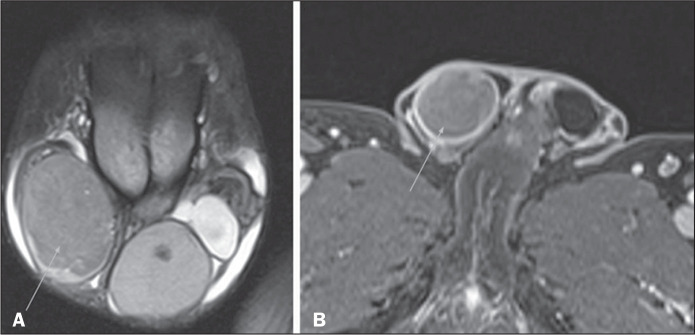
Right testicular seminoma. Coronal T2- weighted image with fat suppression (A) and contrast-enhanced T1-weighted image with fat suppression (B), showing a nodular formation with intermediate to reduced signal intensity on the T2-weighted image (A), hypovascular contrast enhancement (B), and restricted diffusion (image not available). MRI is useful in the preoperative assessment of the local stage of testicular neoplasms, especially in surgical procedures aimed at preserving the testis^(1,4)^. Knowing the size of the tumor, recognizing the potential for invasion of the rete testis or paratesticular space, and identifying a pseudocapsule to facilitate possible tumor enucleation are crucial in this context^(4)^. Smaller seminomas tend to be more homogeneous than are larger lesions, which usually present intervening areas of fluid, with limited differentiation from nonseminomatous GCTs.

The imaging characteristics of testicular neoplasms, such as classic seminomas (Figure 9), are related to their macroscopic and histological appearance^(4)^. These tumors originate from mature cells of the seminiferous tubes and appear as homogeneously solid, lobulated masses with a mixture of tumor cells and fibrous septa infiltrated by lymphocytes and plasma cells. Therefore, they appear as lobulated tumors that are hypointense on T2-weighted images, with hypointense septa appearing prominently on contrast-enhanced sequences^(4)^.

**Figure 10:**
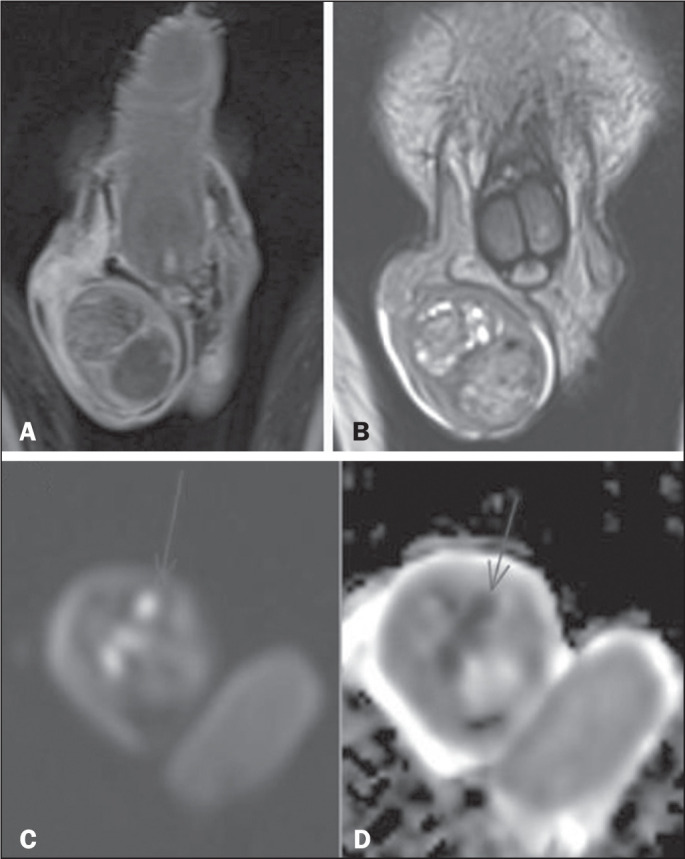
Mixed GCT: 30% choriocarcinoma; 30% endodermal sinus tumor; 20% embryonal carcinoma; and 20% postpubertal teratoma. Contrast-enhanced coronal T1-weighted image (A), high-resolution coronal T2-weighted image (B) and diffusion-weighted images (C,D), showing two heterogeneous nodular formations in the parenchyma of the right testicle, with foci of restricted diffusion.

Nonseminomatous GCTs originate from primitive germ cells and present diverse histological aspects. On MRI, they appear as heterogeneous masses with areas of hemorrhage/ necrosis and varied degrees of contrast enhancement. On T2-weighted images, they can exhibit a hypointense halo, corresponding to the fibrous capsule, although not exclusively^(4)^. In addition, GCTs can also be classified as follows^(9)^: pure (consisting of only one cell type); or mixed (consisting of more than one cell type) (Figure 10).

## CONCLUSION

For evaluating scrotal pathologies, MRI is an excellent imaging method, especially in cases with inconclusive ultrasound findings,^(1,2,4)^ and has gained relevance in recent years, especially because of its more accurate anatomical assessment of structures and contribution to preoperative decision-making. Therefore, there is a growing need to improve the recognition of these pathologies by radiologists, in order to improve diagnostic accuracy and promote appropriate treatment for the affected patients.
